# Antibacterial activities of *Fagara macrophylla, Canarium schweinfurthii, Myrianthus arboreus, Dischistocalyx grandifolius* and *Tragia benthamii* against multi-drug resistant Gram-negative bacteria

**DOI:** 10.1186/s40064-015-1375-y

**Published:** 2015-10-01

**Authors:** Jackson A. Seukep, Bonaventure Ngadjui, Victor Kuete

**Affiliations:** Department of Biochemistry, Faculty of Science, University of Dschang, Dschang, Cameroon; Department of Organic Chemistry, Faculty of Science, University of Yaoundé I, Yaoundé, Cameroon

**Keywords:** Antibacterial activity, *Fagara macrophylla*, *Canarium schweinfurthii*, *Myrianthus arboreus*, Gram-negative bacteria, Multidrug resistance

## Abstract

Bacterial infections caused by multidrug resistant phenotypes constitute a worldwide health concern. The present study was designed to evaluate the in vitro antibacterial activities of the methanol extracts of five medicinal plants: *Fagara macrophylla, Canarium schweinfurthii, Myrianthus arboreus, Dischistocalyx grandifolius* and *Tragia benthamii* against a panel of 28 multidrug resistant Gram-negative bacterial strains. The liquid broth microdilution was used to determine the minimal inhibitory concentration (MIC) and minimal bactericidal concentration (MBC) of the extracts. The best activity was recorded with *Canarium schweinfurthii* bark extract, MIC values ranging from 32 to 1024 µg/mL being recorded against 85.7 % tested bacteria. Broad spectra of antibacterial activities were also obtained with both bark and leaf extracts from *Myrianthus arboreus* (78.6 %) as well as the bark extract from *Fagara macrophylla* (75.0 %). The lowest MIC value of 32 µg/mL was obtained with *Canarium schweinfurthii* bark extract against *Klebsiella pneumoniae* KP63 strain. The results of this work provide baseline information for the use of the studied plants, and mostly *Fagara macrophylla, Canarium schweinfurthii* and *Myrianthus arboreus* in the treatment of bacterial infections including multidrug resistant phenotypes.

## Background

The spread of multidrug resistant bacteria constitutes a major hurdle in chemotherapy (Kuete 2013). In Gram-negative bacteria, efflux pumps belonging to the resistance-nodulation-cell division (RND) family of tripartite efflux pumps are largely involved in multidrug resistance (Van Bambeke et al. 2006). The propagation of bacterial MDR phenotypes is a great challenge for scientist for the discovery of novel antibacterial agents. The role of medicinal plants as sources of anti-infective compounds has been largely documented (Cowan 1999; Kuete 2013; Ndhlala et al. 2013; Ngameni et al. 2013). It was reported that up to 80 % of the world population rely on plants or derived products for their treatment (WHO 1993). Several African medicinal plants previously displayed good antibacterial activities against Gram-negative MDR phenotypes. Some of them include *Dichrostachys glomerata,Beilschmiedia cinnamomea* and *Olax subscorpioïdea* (Fankam et al. 2011), *Lactuca sativa, Sechium edule, Cucurbita pepo* and *Solanum nigrum* (Noumedem et al. 2013b), *Piper nigrum* and *Vernonia amygdalina* (Noumedem et al. 2013a), *Beilschmiedia obscura* and *Peperomia fernandopoiana* (Fankam et al. 2014), *Capsicum frutescens* (Touani et al. 2014), *Fagara tessmannii* (Tankeo et al. 2015). In our ongoing investigation of antibacterial plants, we designed the present work to investigate in vitro antibacterial activity of the methanol extracts of five medicinal plants, *Canarium schweinfurthii Engl*. (Burseraceae), *Dischistocalyx grandifolius C. B. Clarke* (Acanthaceae), *Fagara macrophylla (Oliv.) Engl.* (Rutaceae), *Myrianthus arboreus P. Beauv.* (Moraceae) and *Tragia benthamii Bak.* (Euphorbiaceae) (Table 1) against MDR Gram-negative bacteria.

**Table 1:** Information on the studied plants

Plants samples (family) and Herbarium Voucher number^a^	Part used and extraction yield (%)^b^	Area of plant collection	Traditional treatment	Bioactive (or potentially active) compounds isolated from plants	Biological activities of crude extract^c^
*Canarium schweinfurthii* Engl. (Burseraceae) 19652/HNC	Bark (7.36 %)	Bangangté, West Region of Cameroon	Insecticide, dysentery, gonorrhea, cough, chest pains, pulmonary affections, stomach complaints, food poisoning, purgative and emetic, roundworm infections and other intestinal parasites,emollient, stimulant, diuretic, skin-affections, eczema, leprosy, ulcers (Orwa et al. 2009); diabetes mellitus (Kouambou et al. 2007); colic, stomach pains, gale (Berhaut 1974); fever, constipation, malaria, sexual infection and rheumatism (Koudou et al. 2005)	Essential oil: limonene, phellandrenes (Orwa et al. 2009), triterpenes steroids, terpenoïdes, saponins, tannins, phenolics compounds, alkaloids (Kouambou et al. 2007; Tamboue et al. 2000)	Chemoprevention of cancer (Atawodi 2010); antimycobacterial activities (Nvau et al. 2011); antimicrobial activities against GIPAB (Moshi et al. 2009)
*Dischistocalyx grandifolius* C. B. Clarke (Acanthaceae) 27646/SRFC-Cam	Whole plant (4.53 %)	Bamboutos Mountain, West Region of Cameroon	Fungal and viral infections, cancer, inflammation, anti-pyretic, antioxidant, insecticidal, hepatoprotective, immunomodulatory, Anti- platelet aggregation (Awan and Aslam 2014)	Flavonoids, benzonoids, phenolic compounds, naphthoquinone and triterpenoids (Awan and Aslam 2014)	Not reported
*Fagara macrophylla* (Oliv.) Engl. (Rutaceae) 6173/SRFC-Cam	Leaves (6.81 %) Bark (8.43 %)	Bamboutos Mountain, West region of Cameroon	Malaria (Zirihi et al. 2007); hypertension (Fézan et al. 2008)	Alkaloids: tembetarine,oblongine, magnoflorine, arborinine, nitidine (Torto and Mensah 1970; Tringali et al. 2001); dihydronitidine (Zirihi et al. 2007); acridone alkaloid and amide alkaloids (Wansi et al. 2009); flavonoid: hesperidin (Tringali et al. 2001)	Antiplasmodial activities of ethanol bark extracts (Zirihi et al. 2007); antifeedant activities of isolated acridone alkaloid, arborinine, tembetarine and magnoflorine against *SF*, *SL, SFr* (Tringali et al. 2001)
*Myrianthus arboreus* P. Beauv. (Moraceae) 55499/HNC	Bark (7.68 %) Leaves (10.37 %)	Bangangté, West Region of Cameroon	Dysentery, diarrhea,vomiting; analgesic, antipyretic, heart troubles, pregnancy complications, dysmenorrheal, incipient hernia, boils, toothache, bronchitis, sore throat; headaches, swellings and tumours, diabete (Orwa et al. 2009);stomach disorders (Agwa et al. 2011; Uzodimma 2013)	Alkaloids, flavonoid, tannin (Orwa et al. 2009); cyanogenic glycosides, phytic acid (Agwa et al. 2011); terpenes (Borokini and Omotayo 2012); saponin, anthocyanin, glycoside, carotenoid, oxalate (Otitoju et al. 2014)	Antibacterial activities of methanol and aqueous extracts against KP, PV, SA, EC (Agwa et al. 2011); antiplasmodial activities by inhibiting the developmental stage of AG (Akinkurolere et al. 2011)
*Tragia benthamii* Bak. (Euphorbiaceae) 23329/SRFC-Cam	Whole plant (5.18 %)	Bangangté, West Region of Cameroon	Cough (Oladosu et al. 2013)	Tannins, saponins, flavonoids, alkaloids, (Oladosu et al. 2013)	Antimalarial activity (Oladosu et al. 2013)

^a^(HNC): Cameroon National Herbarium; (SRF/Cam): Société des Réserves Forestières du Cameroun

^b^The percentage of the methanol extract

^c^Microorganisms [SF: *Spodoptera frugiperda*; SL: *Spodoptera littoralis*; SFr: *Spodoptera frugiperda*; KP: *Klebsiella pneumoniae*; PV: *Proteus vulgaris*; SA: *Staphylococcus aureus*; EC: *Escherichia coli*; AG: *Anopheles gambiae*; GIPB: gastrointestinal pathogenic bacteria]

## Methods

### Plant material and extraction

The plants used in this work were collected in different localities of the West Region of Cameroon in January to April 2012. The plants were identified at the National herbarium (Yaounde, Cameroon) where voucher specimens were deposited under the reference numbers (Table 1). Each plant sample was air dried at 24 ± 2 °C, powdered (using a grinder) and a portion of each sample (200 g) was extracted with methanol (MeOH; 1 L) for 48 h at room temperature. The extract was then concentrated under reduced pressure to give residues which constituted the crude extract. All extracts were then kept at 4 °C until further use.

### Antimicrobial assays

#### Chemicals for antimicrobial assay

Chloramphenicol (CHL), (Sigma-Aldrich, St Quentin Fallavier, France) was used as a reference antibiotic (RA). *p*-Iodonitrotetrazolium chloride (INT) was used as microbial growth indicator (Eloff 1998; Mativandlela et al. 2006).

### Microbial strains and culture media

Test organisms included sensitive and resistant strains of *Pseudomonas aeruginosa, Klebsiella pneumoniae, Enterobacter aerogenes, Escherichia coli* and *Providencia stuartii* obtained from the American Type Culture Collection (ATCC) (Lacmata et al. 2012; Seukep et al. 2013). Nutrient agar was used for the activation of the Gram-negative bacteria while the Mueller–Hinton Broth was used for antibacterial assays (Kuete et al. 2011b).

### INT colorimetric assay for MIC and MBC determinations

MIC determinations were conducted using the rapid *p*-iodonitrotetrazolium chloride (INT) colorimetric assay according to described methods (Eloff 1998) with some modifications (Kuete et al. 2008b, 2009). The test samples and RA were first of all dissolved in DMSO/Mueller–Hinton Broth (MHB) broth. The final concentration of DMSO was lower than 2.5 % and did not affect the microbial growth (Kuete et al. 2007, 2008a). The assay was repeated thrice. Wells containing adequate broth, 100 µL of inoculum and DMSO to a final concentration of 2.5 % served as negative control. The MIC of samples was detected after 18 h incubation at 37 °C, following addition (40 µL) of 0.2 mg/mL of INT. MIC was defined as the sample concentration that prevented the color change of the medium and exhibited complete inhibition of microbial growth (Eloff 1998). The MBC was determined by adding 50 µL aliquots of the preparations, which did not show any growth after incubation during MIC assays, to 150 µL of adequate broth. These preparations were incubated at 37 °C for 48 h. The MBC was regarded as the lowest concentration of extract, which did not produce a color change after addition of INT as mentioned above (Kuete et al. 2008b, 2009).

## Results and discussion

The results the antibacterial assays as determined by broth microdilution are summarized in Table 2. Its appears that the tested extracts displayed selective antibacterial activities. The best activity was recorded with *Canarium schweinfurthii* bark extract, the obtained MIC values being ranged from 32 to 1024 µg/mL against 24 of the 28 (85.7 %) test bacteria. Broad spectra of antibacterial activities were also obtained with both bark and leaves extracts from *Myrianthus arboreus* [22/28 (78.6 %)] as well as the bark extract from *Fagara macrophylla* [21/28 (75.0 %)]. MIC values below or equal to 1024 µg/mL were noted with *Fagara macrophylla* leaves and whole-plant extracts from *Dischistocalyx grandifolius* and *Tragia benthamii* on respectively against 13/28(46.4 %), 12/28 (42.9 %) and 11/28 (39.3 %) tested bacteria. The lowest MIC value of 32 µg/mL was obtained with *Canarium schweinfurthii* bark extract against *Klebsiella pneumoniae* KP63 strain. MIC values lower than that obtained for the reference antibiotic chloramphenicol were recorded for *Fagara macrophylla* bark extract against *Enterobacter aerogenes* EA27 (64 µg/mL) and *Canarium schweinfurthii* bark extract (32 µg/mL) against *K. pneumoniae* KP63. The results presented in Table 2 also show that all extracts displayed poor bactericidal effect.

**Table 2:** MICs and MBCs (in μg/mL) of methanol extracts from the studied plants and chloramphenicol

Bacterial strains	Tested samples, MIC and MBC (in bracket) values							
	*Fagara macrophylla*		*Canarium schweinfurthii*	*Myrianthus arboreus*		*Dischistocalyx grandifolius*	*Tragia benthamii*	Reference drug
	B	L	B	B	L	WP	WP	CHL
*Escherichia coli*								
ATCC10536	256 (–)	*64* (1024)	512 (–)	512 (–)	512 (–)	1024 (–)	1024 (1024)	16 (32)
W 3110	1024 (–)	–	1024 (–)	512 (–)	1024 (–)	–	–	64 (128)
MC4100	1024 (–)	–	512 (–)	128 (512)	1024 (–)	512 (–)	1024 (–)	128 (128)
AG100 A	–	–	1024 (–)	256 (1024)	–	1024 (–)	1024 (–)	64 (64)
AG100Atet	512 (–)	1024 (–)	1024 (–)	256 (–)	1024 (–)	512 (–)	–	64 (128)
AG102	256 (1024)	512 (–)	512 (–)	512 (–)	256 (–)	–	–	64 (128)
AG100	512 (–)	512 (1024)	1024 (1024)	1024 (–)	256 (–)	–	–	16 (64)
*Enterobacter aerogenes*								
ATCC13048	1024 (–)	–	1024 (–)	1024 (–)	256 (–)	–	–	8 (32)
EA294	1024 (–)	–	1024 (–)	512 (1024)	256 (–)	1024 (–)	**–**	16 (128)
CM64	1024 (–)	1024 (–)	–	1024 (–)	–	–	1024 (–)	128 (–)
EA298	1024 (–)	–	1024 (–)	–	512 (–)	–	1024 (–)	256 (–)
EA27	*64* (512)	256 (512)	512 (–)	128 (1024)	256 (–)	512 (–)	256 (1024)	–
EA289	–	–	–	512 (–)	1024 (–)	–	–	256 (–)
EA3	1024 (–)	1024 (–)	512 (–)	512 (–)	512 (–)	1024 (–)	–	–
*Klebsiella pneumoniae*								
ATCC11296	1024 (–)	–	1024 (–)	1024 (–)	1024 (–)	–	**–**	8 (256)
KP55	1024 (–)	512 (–)	1024 (–)	512 (–)	512 (–)	–	–	32 (128)
KP63	256 (1024)	512 (–)	*32* (512)	128 (512)	256 (512)	512 (–)	1024 (–)	128 (–)
K2	1024 (–)	512 (–)	512 (–)	512 (–)	512 (–)	–	–	64 (256)
K24	1024 (–)	–	512 (–)	1024 (–)	1024 (–)	–	–	32 (256)
*Pseudomonas aeruginosa*								
PA01	–	–	–	–	–	–	–	128 (–)
PA124	–	–	1024 (–)	–	–	–	–	256 (–)
*Providencia stuartii*								
ATCC29916	1024 (–)	1024 (–)	1024 (–)	512 (–)	256 (–)	512 (–)	1024 (–)	16 (32)
PS2636	1024 (–)	1024 (1024)	256 (–)	–	512 (1024)	–	1024 (–)	32 (32)
PS299645	–	–	1024 (–)	1024 (–)	512 (–)	–	–	32 (256)
NEA16	512 (1024)	1024 (–)	512 (–)	256 (1024)	512 (–)	256 (–)	256 (512)	256 (–)
*Enterobacter aerogenes*								
BM47	1024 (–)	–	512 (–)	–	1024 (–)	–	–	256 (–)
ECCI69	–	–	512 (1024)	–	–	1024 (–)	1024 (–)	–
BM67	–	–	–	1024 (–)	–	1024 (–)	–	256 (–)

(–): >1024 µg/mL for plants’ extracts and >256 µg/mL for chloramphenicol (CHL). In italics: significant activity (Kuete 2010; Kuete and Efferth 2010)

*Ec* *Escherichia coli*, *Ea* *Enterobacter aerogenes*, *Kp* *Klebsiella pneumoniae*, *Pa* *Pseudomonas aeruginosa*, *Ps* *Providencia stuartii*, *Ecl* *Enterobacter cloacae*, *B* bark extract, *L* leaves extract, *WP* whole plant extract

Several molecules belonging to classes of secondary metabolites previously reported in the tested plants (Table 1) have been reported to be active on pathogenic microorganisms (Awouafack et al. 2013; Cowan 1999; Ndhlala et al. 2013; Tsopmo et al. 2013). The presence of such metabolites in our extracts could explain their antibacterial activities. According to Kuete (2010), Kuete and Efferth (2010), the antibacterial activity of a plant extract is considered significant when the MICs are below 100 μg/mL, moderate when 100 ≤ MIC ≤ 625 μg/mL and weak if MIC >625 μg/mL. Consequently, the activity of *Fagara macrophylla* bark extract against *Escherichia coli* ATCC10536 and *Enterobacter aerogenes* EA27 and (MIC of 64 µg/mL) and *Canarium schweinfurthii* bark extract against *K. pneumoniae* KP63 (MIC of 32 µg/mL) can be considered important. The MIC values reported herein for the studies plants and mostly *Fagara macrophylla, Canarium schweinfurthii* and *Myrianthus arboreus* are moderate in general but can be considered important when regarding the medicinal importance of the tested MDR bacteria (Chevalier et al. 2000; Kuete et al. 2010, 2011a; Mallea et al. 1998, 2003; Pradel and Pages 2002; Tran et al. 2010). The antimicrobial properties compounds from *Canarium schweinfurthii* have been reported (Longanga Otshudi et al. 2000); also, the antibacterial activity of *Myrianthus arboreus* was also reported against *Klebsiella pneumoniae, Proteus vulgaris*, *Staphylococcus aureus* and *Escherichia coli* (Agwa et al. 2011). The present study provides additional data on the ability of this plant to fight MDR bacteria of these plants as well as information on the antibacterial potentcy of other extracts.

## Conclusion

The results of this work suggest that the studied plant extracts, particularly those from *Fagara macrophylla, Canarium schweinfurthii* and *Myrianthus arboreus*, can be used to control some infections and especially those involving MDR bacterial species. Full purification of this plants in the future will be achieved to identified their antibacterial constituents.
